# Cell organelles are retained inside pyroptotic corpses during inflammatory cell death

**DOI:** 10.1042/BSR20231265

**Published:** 2023-10-27

**Authors:** Anne Hempel, Andrea D'Osualdo, Scott J. Snipas, Guy S. Salvesen

**Affiliations:** Sanford Burnham Prebys Medical Discovery Institute, La Jolla, CA 92037, U.S.A.

**Keywords:** caspases, cytokines, Gasdermin, inflammasome, Membrane pore

## Abstract

Many proinflammatory proteins are released via the necrotic form of cell death known as pyroptosis. Sometimes known as gasdermin D (GSDMD) dependent cell death, pyroptosis results from the formation of pores in the plasma membrane leading to eventual cell lysis. Seeking to understand the magnitude of this cell lysis we measured the size of proteins released during pyroptosis. We demonstrate that there is no restriction on the size of soluble proteins released during pyroptosis even at early timepoints. However, even though large molecules can exit the dying cell, organelles are retained within it. This observation indicates that complete cell rupture may not be a consequence of pyroptosis, and that plasma membrane architecture is retained.

## Introduction

Pyroptosis is a regulated cell death mechanism mostly found in professionally phagocytic cells such as macrophages, monocytes, and dendritic cells [1]. It is a prominent effector arm of the innate immune system in vertebrates that reacts to infection through detection of pathogen-associated molecular patterns (PAMPs). Pyroptosis results in the rupture of the affected cell membrane, thereby releasing inflammatory cytokines and damage-associated molecular patterns (DAMPs) and priming adaptive immunity.

Induction of pyroptosis leads to the activation of inflammatory caspases which in turn process the pyroptotic effector protein gasdermin D (GSDMD) [2,3]. The GSDMD N-terminal fragment then oligomerizes to form pores in the plasma membrane [4–6], leading to the release of cytosolic DAMPs that induce tissue inflammation and recruit the adaptive immune system [7].

Pyroptosis can be subdivided into the canonical and non-canonical pathways [8]. In canonical pyroptosis, DAMPs are detected by specific pattern recognition receptors. Upon binding of a danger signal those proteins assemble into multimeric structures called inflammasomes, sometimes called pyroptosomes [9]. Inflammasomes can either recruit and activate caspase-1 directly or through association with the adapter protein apoptosis associated speck-like protein containing CARD (ASC). In non-canonical pyroptosis intracellular pathogens activate caspase-11 (the mouse orthologue of human caspase-4 and -5). Thus, the different pathways can be defined by which specific inflammatory caspase is engaged [10]. Canonical pyroptosis through induction of the NLRP3 inflammasome follows a two-step pathway [11]. The cell is primed through external PAMP recognition of LPS leading to an upregulation of NFkB controlled gene expression, amongst which inflammasome components and pro-inflammatory cytokines are expressed to sufficient levels required for activation. A second signal (notably involving K+ efflux) [11] is then recognized by NLRP3, leading to its oligomerization. The NLRP3 inflammasome requires the adapter ASC for recruitment and activation of caspase-1. Active caspase-1 will process the pro-inflammatory cytokines interleukin (IL)-1b and -18 to their mature forms [8,10]. Caspase-11 cannot process these cytokines directly [12] and requires the downstream activation of caspase-1 to accomplish this task [2,13]. The release of inflammatory cytokines from the cell is therefore readily explained by caspase and GSDMD mediated pore formation from the lysing cell corpse.

Studies using synthetic membranes have suggested that GSDMD pores have an average diameter of 13 nm [4–6], which is sufficient for release of IL-1b which has a diameter of approximately 4.5 nm. Formation of GSDMD pores results in the recruitment and activation of a second lytic molecule Ninjurin 1 (NINJ1) that is required for eventual cell rupture [14].

To gain insight into the effective magnitude of pyroptotic pores, we set out to determine the kinetics of protein release from mouse macrophages undergoing canonical and non-canonical pyroptosis.

## Results

### Pyroptosis leads to loss of membrane integrity, release of soluble cytosolic contents and mature IL-1b in a time-dependent manner

By definition, biomarkers of pyroptosis are processed IL-1b and IL-18 since their accurate processing relies on inflammatory caspases. Because pyroptosis is a lytic cell death mechanism its progression can be monitored biochemically in tissue culture. We utilized parallel approaches to monitor the kinetics of canonical pyroptosis via: (1) vital dye influx into the cell by measuring propidium iodide (PI) staining; (2) enzymatic lactate dehydrogenase (LDH) assay that measures the appearance of the cytosolic enzyme in the cell supernatant; and (3) by evaluating total protein released from the cell (releasates) by Coomassie stained SDS PAGE.

**Figure 1:**
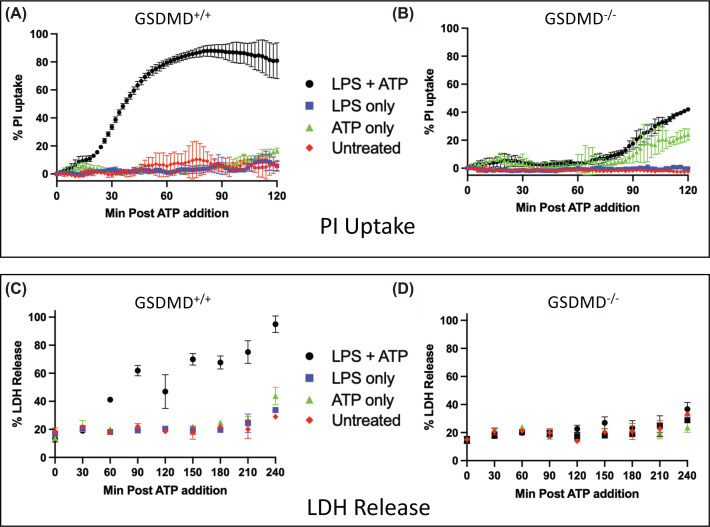
PI uptake and LDH release in murine macrophages RAW 264.7 cells ectopically expressing ASC were primed with LPS and activated with ATP as described in methods. PI uptake was analyzed with continuous fluorescent measurements. (**A**) PI uptake assay of RAW 264.7 cells containing GSDMD. (**B**) Same assay using GSDMD-/- cells. LDH activity was assayed using a discontinuous assay measuring released LDH to the cell culture supernatant over 4 h with samples taken every 30 min. (**C**) LDH release of RAW 264.7 cells containing GSDMD. (**D**) Same assay using GSDMD-/- cells. Data represent mean and SD of three technical replicates.

As a model cell line to examine pyroptosis we utilized the murine macrophage cell line RAW 264.7. Because this line is naturally deficient in ASC we reconstituted with ASC (see methods). Cells were primed with LPS and the NLRP3 inflammasome activated with ATP, both applied in the extracellular environment, followed by analysis of the kinetics of canonical pyroptosis and how GSDMD influences its progression. A loss of membrane integrity could be seen as early as 10 min post ATP, revealed by influx of PI and efflux of LDH (Figure 1). GSDMD protein was deleted from RAW ASC cells through CRISPR/Cas9, revealing that the early phase of release was dependent on GSDMD since GSDMD^−/−^ cells now show a delay in PI uptake and do not release LDH.

### Loss of Gasdermin D prevents membrane integrity loss, release of cellular components and mature IL-1b at early time points

**Figure 2:**
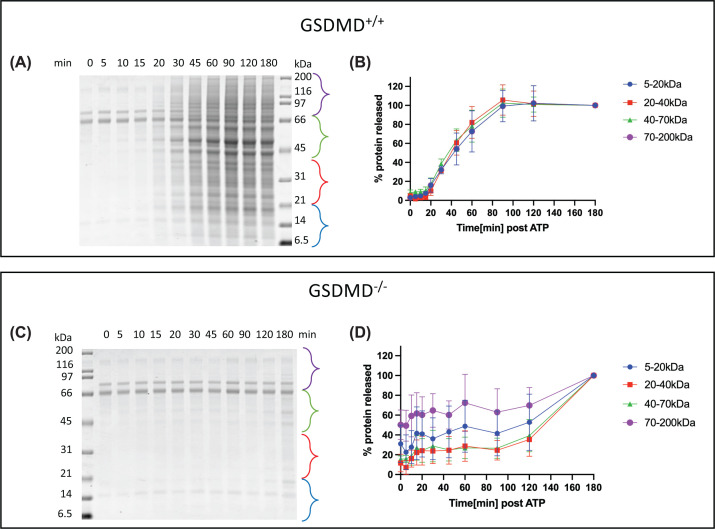
Time course and size dependence of protein release from pyroptotic cells RAW 264.7 ectopically expressing ASC were primed with LPS and activated with ATP as described in methods. Supernatants were collected, TCA precipitated and analyzed by SDS-PAGE, with staining by Coomassie blue (Panels **A** and **C**). Areas represented by the brackets for each timepoint were selected for quantitation by densitometry (Panels **B** and **D**). Released protein was normalized considering the 180-min timepoint as 100%. Data represent mean and SD of gels run in triplicate from three independent experiments.

The kinetics of total protein release following LPS/ATP treatment showed a sigmoidal relationship, dependent on GSDMD (Figure 2). The apparent background release of proteins from GSDMD^−/−^ cells is a consequence of normalizing to the 180-min time point (Figure 2C,D). The releasates show a homogenous increase over all protein sizes over time. Quantification of released protein on the gel in size ranges from 5 to 20 kDa, 20 to 40 kDa, 40 to 70 kDa and 70 to 200 kDa shows no difference in the amount that was released at any time points. This independence of protein size would indicate that pores formed by GSDMD lead to a quick overall lysis of the cell, therefore not limiting the size of proteins that can exit the cell.

### Cellular compartments are not compromised during pyroptosis

**Figure 3:**
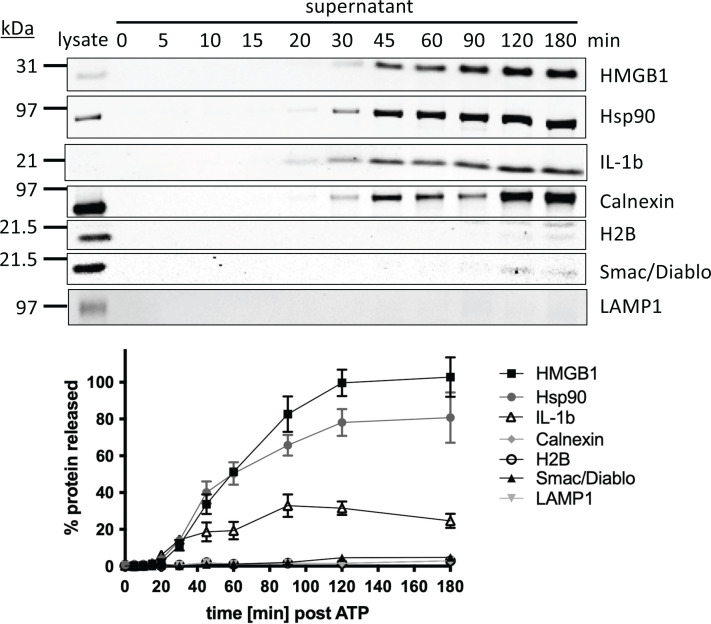
Time course of release of organelle marker proteins from pyroptotic cells RAW 264.7 ectopically expressing ASC were primed with LPS and activated with ATP as described in methods. Supernatants were collected, TCA precipitated and analyzed by SDS-PAGE (as in Figure 2). Note that only 5% of the lysate sample (from untreated cells) was loaded on to the gel. This serves as the total protein for normalization purposes. Following transfer to nitrocellulose proteins were detected by antibodies specific to the indicated proteins. Band intensity was normalized to the cell lysate (first lane). Data represent mean and SD of gels run in triplicate from three independent experiments.

**Figure 4:**
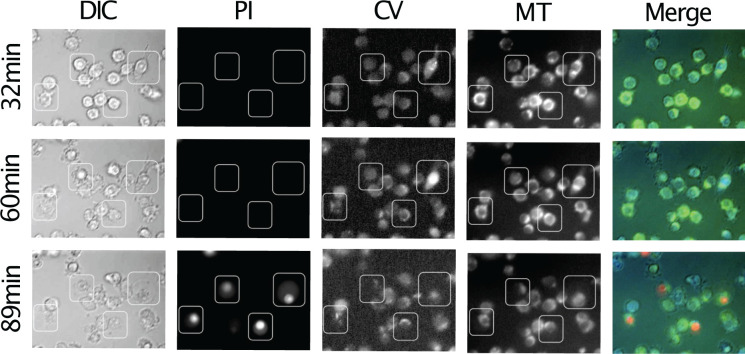
Timelapse images of pyroptotic cells RAW 264.7 cells were stimulated with LPS and ATP to undergo canonical pyroptosis and monitored by fluorescent microscopy. Live cell cytoplasm was revealed by calcein violet (CV) and mitochondria by mitotracker (MT). Pyroptosis was monitored by the uptake of propidium iodide (PI). The images reveal that cells become permeable to small molecular weight dyes, while retaining intracellular organelles.

When examining the proteins released during pyroptotic lysis of the cell we found no release of intra-organelle proteins (Figure 3). Interestingly, mature IL1b release seemed to plateau at approximately 90 min, possibly because caspase-1 and, or the inflammasome are lost from cells [15]. Proteins characteristic of the major cellular organelles (H2B, nucleus; SMAC/Diablo, mitochondrion; LAMP-1, lysosome) reveal no exit of these organelles from pyroptotic cells within the 3-h time course of the experiment (Figure 3). This leads to the hypothesis that even though there is swelling of the cell and lytic release of proteins, some clear membrane boundary is conserved. In support of this, lysing cells treated with compartmental marker dyes, retain their organelle-specific fluorescence whilst loosing unspecific cytosolic dye just before the influx of the membrane impermeable dye propidium iodide (Figure 4).

## Discussion

**Figure 5:**
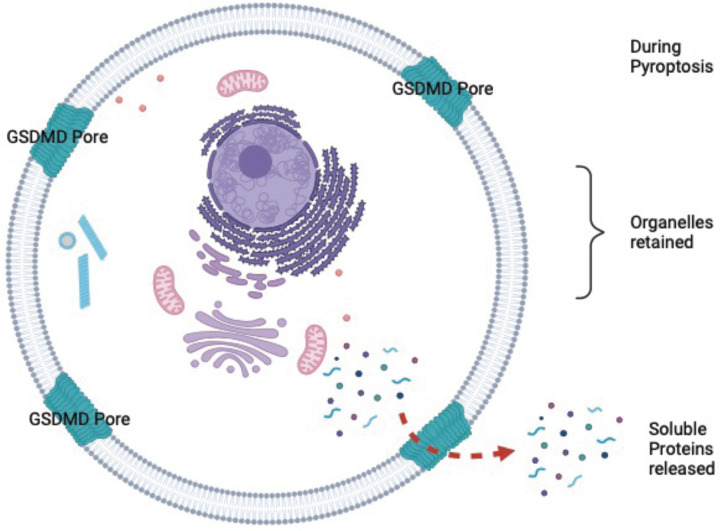
Figure illustrating the main conclusions of the study Figure rendered with the help of BioRender.com

The release of the mature form of the key pro-inflammatory mediators IL1b and IL18 from macrophages has been known for many years to be dependent on cleavage by caspase-1. Both are among a group of IL1 family members that lack conventional secretion signals. Some of them (such as IL1b and IL18) require caspase cleavage and some (such as IL1a) don’t and thus there had been a puzzle, only recently resolved, about how these cytokines were released. Genetic evidence combined with chemical inhibitors has revealed the key mediator of this release to be GSDMD, which causes a cell lytic event upon cleavage by inflammatory caspases 1 and 11. Thus the macrophage must die in order to release pro-inflammatory mediators. This lytic death contrasts starkly with apoptosis, the other caspase-mediated cell death, which does not result in membrane rupture and release of cellular contents [8,10]. Working with synthetic membranes, the number of GSDMD N-terminal monomers that make up the pore seems to be flexible to a certain extent, with varying reports from 16 [5] to 24 [4] subunits, an observation that has also been reported for pore forming complexes such as Complement, Perforin, and some bacterial pore-forming toxins [16]. Most GSDMD pores ranged in diameter from 10 to 20 nm, with an average of approximately 13 nm [3,4,6,17]. Pores of this diameter are large enough to permit efflux of large proteins and protein complexes up to approximately 2.5 MDa (assuming an upper limit of the pores of 20 nm) but would restrict egress of larger intracellular components. The artificial membrane experiments seem to match our observation in an experimental cellular system. Soluble proteins over a large molecular weight range are released, yet cellular organelles are retained. Our data are consistent with the observation that activation of the noncanonical pyroptosis pathway leads to the release of many soluble cytosolic proteins, with no organelle associated proteins reported [14]. Eventual GSDMD-dependent release of cytosolic proteins is ultimately reliant on the protein ninjurin-1 (NINJ1), that is itself implicated in plasma membrane rupture [14]. Our data reveal that such rupture does not result in the release of organelles, only soluble cytosolic proteins (Figure 5). Because IL1b release plateaus at 90 min, after the release of most proteins (Figure 3), we conclude that caspase dependent activation of this inflammatory mediator is slower than activation of the lytic function of GSDMD.

Pore formation is reversible, and eukaryotic cells can repair plasma membrane lesions via various mechanisms, primarily centered around endocytic and exocytic pathways [18,19]. Indeed, pores triggered by GSDMD activation can be repaired to reinstate cell membrane integrity [20,21]. A competition between pore formation and pore repair thus defines the kinetics and extent of release of cytosolic contents from pyroptotic cells.

## Materials and methods

### RAW264.7-Asc cells generation

The coding sequence of mouse Asc was cloned in the pRDI292 lentivector and lentiviral particles were generated in HEK293T by co-transfection with packaging vectors pMD2.G and pCMV-dR8.91 (plasmids were kind gift from Jurg Tschopp). After transduction, cells were selected with 5ug/ml puromycin and Asc expression was verified by Western blotting.

### LDH release assay

Lactate dehydrogenase (LDH) is a cytosolic enzyme that is released via cell membrane lysis during cell death *in vitro*. Cells were seeded at 5 × 10^4^ cells/well. Upon treatment and incubation of cells with death-inducing treatments, cell culture supernatants were centrifuged for 5 min at 3,000 × ***g*** and 4°C, to remove cell debris. For LDH enzymatic reactions, 50 μl of supernatants were mixed with 50 μl of LDH substrate mix from the CytoTox 96 Non-Radioactive Cytotoxicity Assay (Promega) in 96-well plates and incubated for 30 min at room temperature. Then, 50 μl of stopping solution was added to the reactions and mixed with a pipette. Absorbance was recorded at 492 nm. Untreated cells were lysed with 10× lysis solution included in the assay kit and used to normalize the amount of released LDH.

### Propidium iodide uptake assay

RAW 264.7 ectopically expressing ASC were seeded at 1 × 10^5^ cells/well in a 96-well black plate. To prime the cells 100 ng/ml LPS in cell culture media was added to the appropriate wells with and incubated at 37°C 5% CO_2_ for 4 h. Media were removed and cells were washed in PBS. Cells were then activated in Optimem with or without 5 mM ATP. PI (3 μM) was added to each well and continuous fluorescent measurements were monitored for 2 h using a CLARIOstar plate reader (BMG LabTech) at excitation 535–620 nm and emission at 618–630, at a gain of 2416 and focal height of 4.0. Percent of PI uptake was normalized to fluorescence emitted by completely lysed cells obtained by submitting the plate to 3 freezing at −80°C and thawing at 37°C cycles at the end of the experiment.

### Preparation of samples

Cells were subjected to death stimuli, and supernatants were collected at indicated time points. Supernatants were centrifuged to remove cellular debris and transferred into a clean tube for analysis. Remaining cells were harvested and washed in PBS. Cells were lysed directly in 1× SDS gel loading buffer and analyzed. Trichloroacetic acid (TCA) protein precipitation was used to concentrate protein samples or to remove contaminants, including salts like citrate, prior to applications like protein electrophoresis and Western blotting. Addition of the following components is determined considering the starting sample volume. Protein was precipitated by addition of 1/2 sample volume of 30% TCA, followed by vortexing and centrifugation at 16,000 × ***g*** for 30 min at 4°C. The supernatant was discarded and 1 sample volume of 10% TCA was added to pelleted protein. Sample was centrifuged at 16,000 × ***g*** for 10 min at 4°C. The supernatant was discarded, and the pellet was washed two times with 2 sample volumes of pure acetone, followed by centrifugation for 2 min at 16,000 × ***g***. Pellets were dried at room temperature and proteins were resuspended in 20 μl of SDS-PAGE sample buffer.

### SDS-PAGE

Sodium dodecyl-sulfate polyacrylamide gel electrophoresis (SDS-PAGE) was used to separate proteins according to their apparent molecular weight. Proteins were premixed with SDS-PAGE sample buffer and heated at 95°C for 5 min. Pre-cast Bolt 4-12% Bis-Tris Plus gels (Invitrogen) were run at constant 165 or 200 V, for 25–35 min using NuPAGE SDS Running MES Buffer. Gels were washed briefly with milli-Q water and stained with InstantBlue Coomassie stain (Expedeon) for at least 1 h, followed by destaining with milli-Q water or used directly for Western blotting.

### Western blot

To further analyze proteins separated by SDS-PAGE, they were transferred to nitrocellulose membrane using wet blotting and followed by immunodetection. Proteins were transferred in Bolt Transfer Buffer (Life Technologies) for 1 h at room temperature and 10 V. To verify that protein transfer was successful and uniform, membranes were stained with Ponceau solution for 1 min and distained in water until pink bands on a white background were revealed. Membranes were stained with Ponceau followed by washing with Tris Buffered Saline (TBS) for 5 min to remove the Ponceau solution, and then incubated in 5% BSA TBS-Tween (TBS-T) under continuous shaking for 1 h at room temperature to block unspecific binding sites. Incubation with the primary antibody was carried out in 5% BSA TBS-T at the appropriate dilution overnight under continuous shaking at 4°C. To remove unbound primary antibody, membranes were washed three times for 5 min with TBS-T at room temperature. The secondary antibody was diluted in TBS-T and incubated for 1 h before repeating the washing steps. Detection was performed using an Odyssey CLx imaging equipment (LI-COR Biosciences).

### Densiometric analysis of protein releasates

Supernatants taken from RAW 264.7 cells undergoing pyroptosis were run on SDS-PAGE, stained by Coomassie, or transferred by Western blotting. Bands were visualized by staining or detected by antibodies specific to the proteins of interest. Detection and quantitation were performed using an Odyssey CLx imager (LI-COR Biosciences).

### Gasdermin KO

RAW 264.7 cells expressing ectopic ASC were transfected with plasmids containing Cas9 and the guide RNA targeting exon 4 of GSDMD. The guide RNA with highest target specificity was selected using the CRISPR design tool from MIT (http://crispr.mit.edu). Each gRNA was cloned in the pX330-U6-Chimeric_BB-CBh-hSpCas9 plasmid, a gift from Feng Zhang (Addgene plasmid #42230). Cells were single-cell sorted 6h after transfection and grown until colonies were visible. Clones were expanded and screened for GSDMD expression by Western blot with GSDMD antibody (Sigma). Clones without detectable GSDMD protein were sent for sequencing to confirm a stop codon was generated by indel at the appropriate location.

### Microscopy

Cells were seeded the day before the experiment on glass bottom culture plates. The cells were first stained with calcein violet and mitotracker (Invitrogen) for 30 min and then washed to remove the dye. Cells were stimulated with LPS and ATP in the presence of propidium iodide. Images were taken every minute at the appropriate wavelength to track cell death and abundance of fluorescent signals.

### Statistical analysis

Statistical analyses were performed using GraphPad Prism software (version 8.9; GraphPad Software Inc.).

## Data Availability

All data needed to evaluate the conclusion in the paper are present in the paper. Source data of this study are available from the corresponding author upon request.

## Abbreviations

ASC: apoptosis associated speck-like protein containing CARD
DAMP: damage-associated molecular pattern
GSDMD: Gasdermin D
IL: interleukin
LDH: lactate dehydrogenase
NINJ1: Ninjurin 1
PAMP: pathogen-associated molecular pattern
PI: propidium iodide
TCA: trichloroacetic acid
